# Medical Treatment One Hundred Years Ago

**Published:** 1910-06

**Authors:** 


### Medical Treatment One Hundred Years Ago.

The following letter was written by Dr. Rush to a patient who had been to the South for his health and was returning to his family in New York. It is reprinted from *The Boston Medical and Surgical Journal* to whom it came from the descendants of the patient:

Philadelphia, April 15, 1812.

SIR: The following remedies appear to me proper in your case:

1. Garlic taken in substance, that is, a bruised clove three times a day, or in infusion, that is, eight or ten cloves bruised and put into a pint of peppermint tea, of which a wineglassful should be taken three times a day.

2. The oil of amber—of which from ten to fifteen drops should be taken three times a day in a tablespoonful of molasses.

3. A mixture composed of tar, molasses, lime or lemon juice, and old spirit of any kind, of each one gill, ultimately united by simmering for a few minutes over the fire. A teaspoonful of this medicine should be taken three times a day.

The above three medicines should be taken in succession and rotation in the order in which they are mentioned. A month or more may be interposed between the use of each of them.

4. A perpetual blister about the size of a French crown should be opened upon your left arm, between the shoulder and elbow. It should be kept open until your disease is removed.

5. Receive frequently into your lungs the vapor of a quart of boiling water poured upon a gill of tar in a mug through funnel placed over the mug.

6. Take as many drops of laudanum every night as will compose your cough. Continue to take laudanum in the morning. A pill of a grain or two grains of opium may be taken at bedtime if the ordinary dose of laudanum should lose its effect.

7. Go to bed early. Sleep upon a mattress in summer and a feather bed in winter.

8. Wear flannel next to your skin in winter and muslin in summer. Continue your former diet and drinks.

9. Use gentle exercise daily, especially that kind of exercise which employs the arms.

10. Carefully avoid the morning and evening air, also fatigue from all its causes.

11. Should your pulse at any time, from taking cold or any other cause, become tense or pale, and your cough be more troublesome than usual, lose eight or ten ounces of blood, and abstain from animal food and from the use of the first three medicines recommended to you, until your pulse be restored to its ordinary strength. If one bleeding does not reduce your pulse, repeat it until it is reduced.

12. Spend the last month of next autumn, all the winter months and the first and part of the second month in the spring in a large room, warmed by a close stove, not only eat and drink and sit during the day in this room, but sleep in it. Exercise, or gentle labor of any kind may be used in it. Its temperature should be uniformly between 65 degrees and 75 degrees. This remedy is to be used only in case you are not much better before the 1st of November.

13. Should the above remedies fail of relieving you in three months, recourse should be had to a salivation, after which resume them all. Should your family physician advise it, the salivation may be resorted to as soon as you reach home, and before you begin to take any of the medicines herein prescribed, except such as are intended to relieve your cough.

(Signed) BENJ'N. RUSH.

Verily the patient had a fighting chance for life 100 years ago. How kind nature must have wept at the cruel blistering, bleeding and dosing, and this by the famous Dr. Rush, who, in 1910, would have been mobbed. Will a hundred years hence tell the same story of the surgeon's flashing blade of today?—*The Medical Standard.*

